# Follow‐up results regarding fixation of contralateral testis after testicular torsion in children

**DOI:** 10.1002/pdi3.48

**Published:** 2024-04-02

**Authors:** Chengjun Yu, Yi Wei, Qinlin Shi, Sheng Wen, Tao Lin, Dawei He, Guanghui Wei, Shengde Wu

**Affiliations:** ^1^ Department of Urology Children's Hospital of Chongqing Medical University Chongqing China; ^2^ Chongqing Key Laboratory of Structural BirthDefect and Reconstruction Chongqing China; ^3^ National Clinical Research Center for Child Health and Disorders Chongqing China; ^4^ Chongqing Key Laboratory of Pediatrics Chongqing Chongqing China; ^5^ Ministry of Education Key Laboratory of Child Development and Disorders Chongqing China; ^6^ China International Science and Technology Cooperation Base of Child Development and Critical Disorders Chongqing China

**Keywords:** contralateral testicular fixation, pediatrics, testicular torsion

## Abstract

The aim is to investigate the possible advantages and disadvantages of contralateral testicular fixation (CTF) for boys with testicular torsion (TT) by long‐term clinical follow‐up results. A retrospective cohort study was conducted on TT boys at Children’s Hospital of Chongqing Medical University from September 2005 to December 2020. The baseline characteristics, intraoperative findings, and clinical follow‐up results were compared between TT patients underwent orchiopexy with or without CTF, and orchiectomy with or without CTF, respectively. The *χ* ^2^ test and the Fisher’s Exact test were used to compare dichotomous outcomes, and the Mann‐Whitney U test was used to compare continuous outcomes without normal distribution. A total of 140 cases were included in this study. The bell clapper deformity was found in 30.4% of all torsed testes underwent orchiopexy, 16.4% during contralateral testicular exploration, and only 10.9% in bilateral sides. Contralateral testicular volume, adverse events, and paternity rates were comparable between patients underwent orchiopexy with or without CTF, and no subsequent contralateral TT observed during follow‐up. However, those patients underwent orchiectomy without CTF had more incidence of scrotal discomfort than those had orchiectomy with CTF (*P* = 0.027). In addition, one boy suffered subsequent contralateral TT after orchiectomy without CTF. The incidence of bell clapper deformity was lower in this research than that of literature reports. CTF would not induce testicular volume loss, atrophy or scrotal pain. Contralateral testicular discomfort and even subsequent TT could be more easily observed during follow‐up in patients without CTF. We still recommend routine surgical fixation of contralateral testicle during surgery.

## Graphical abstract

Contralateral testicular fixation would not induce testicular volume loss, atrophy, or scrotal pain. Contralateral testicular fixation may decrease the risk of contralateral testicular discomfort and even subsequent testicular torsion. We still recommend routine surgical fixation of contralateral testicle during surgery.

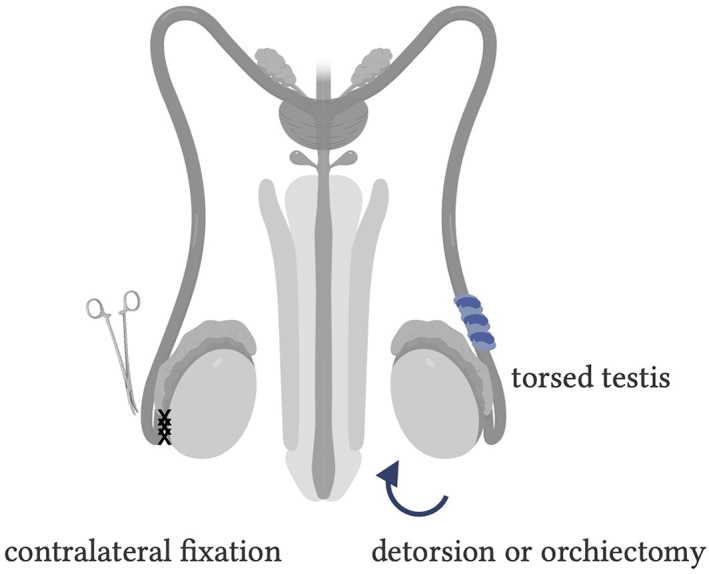

## 1 INTRODUCTION

Testicular torsion (TT) is defined as the tunica vaginalis or the spermatic cord primarily or secondarily twisting along the longitudinal axis, causing testicular blood flow obstruction and subsequent damage.^1^ The exact etiology for TT is not clear yet, but bell clapper deformity (BCD) is associated with an increased risk of intravaginal TT.^2^ It is accepted by urologists that the prenatal or neonatal TT usually be the extravaginal type, while acute TT later in life almost always be the intravaginal type.^2^, ^3^ TT is a urological emergency that needs prompt surgery, because delayed diagnosis and management of TT would result in a higher rate of testicular atrophy or even totally loss of affected testis and eventually impairment of fertility.^4^, ^5^, ^6^ Unfortunately, the latest systematic review of surgical technics on TT still did not find the most suitable one restricted by limited evidence.^7^

According to European Association of Urology guidelines on pediatric urology 2020 (available at https://uroweb.org/guideline/paediatric‐urology), whether performing the prophylactic fixation of contralateral testicle of boys with TT is really necessary still remained as an unanswered question. Performing the contralateral testicular fixation (CTF) undoubtedly greatly affects the decision of surgical technique for TT and is still under a great controversy. In TT patients, CTF was mainly conducted to minimize the risk of catastrophic bilateral testicular loss.^8^ In addition, CTF arouse the concerns about potential risks of iatrogenic damage to the contralateral healthy testicle.^9^

A survey from the United Kingdom conducted by Rhodes and colleagues regarding practice among pediatric surgeons and urologists on neonatal TT found that about 71.9% of surgeons chose to perform the orchiectomy and CTF simultaneously.^10^ Not long after that time, a similar survey from the America found that about 93.0% of surgeons would perform a contralateral orchiopexy.^11^ Almost all the participated pediatric urologists performed CTF in TT cases, though the authors still emphasized the necessity of patients, parents, and surgeons on the decision for CTF, according to the latest survey on current practice patterns of CTF in 2018.^8^ It seemed that more and more pediatric surgeons chose to accept and perform the CTF during prompt surgery for TT, while the real necessity and benefits of CTF and potential damage of CTF on contralateral healthy testicle were still unclear. The lacking of long‐term clinical results on this topic greatly restricted the clinical decision making and guidelines. Therefore, the aim of this study is to compare the clinical long‐term outcomes of pediatric patients with TT between those who received CTF and those who did not, regarding testicular volume (TV), testicular atrophy, adverse events during follow‐up and paternity rate.

## 2 METHODS

### 2.1 Patient populations

This retrospective cohort study was conducted at the Children's Hospital of Chongqing Medical University from September 2005 to December 2020. The local institutional review board of Children's Hospital of Chongqing Medical University approved this study (File No. 2021‐384), and written informed consents were obtained from caregivers for scientific research during hospitalization. The clinical information and follow‐up results were collected from hospital records, operation records, out‐patient medical records, and an extra arranged telephone interview. In the telephone call, patients or caregivers were asked about scrotal‐associated symptoms, including scrotal pain, swelling, redness, or discomfort, and were recommended for a visit for physical examination and ultrasonographic monitoring if possible.

Demographic and clinical data were extracted and data of birth, time of symptoms onset and surgery, age at follow‐up, surgical strategy, intraoperative findings, and follow‐up results were recorded. Inclusion criteria for this study were boys older than 6 months and younger than 18 years with surgically confirmed TT and underwent orchiopexy or orchiectomy with or without CTF and with sufficient follow‐up information at our hospital with duration of more than 1 month. Exclusion criteria were TT with undescended testes, prenatal or neonatal torsion, negative explorations, incomplete follow‐up information (at their local hospital or unwilling to answer scheduled questions), those patients without fixation of affected testis, or those not being able to contact.

### 2.2 Study design and definitions

The baseline characteristics, intraoperative findings, and follow‐up results were compared between these TT patients who underwent salvage orchiopexy with or without CTF and orchiectomy with or without CTF, respectively.

Baseline characteristics included age at surgery, duration from symptom onset to surgery, and laterality. Intraoperative findings included direction of rotation, degree of cord twisting, grade of Arda, and BCD found during exploration. The definition of grade of Arda was derived from the bleeding status of cut surface of testicular tissue during surgery by Arda et al. As grade I, immediate bleeding after incision; grade II, no bleeding immediately but starting <10 min; and grade III, no bleeding until 10 min.^12^ Follow‐up results included age, duration from surgery to follow‐up, TV, adverse events after surgery, ipsilateral testicular atrophy, and paternity rates. TV was calculated according to the formula L × W × H × 0.71,^13^ and testicular length (L), width (W), and height (H) were measured by ultrasound (high frequency, GE vivid E9). Particularly, in comparison of TV, we also matched a healthy referral group according to age and laterality with a ratio of 1:3 from the physical examination center. Adverse events after surgery included subsequent orchiectomy, testicular tumor, contralateral TT, and scrotal pain or discomfort. Testicular atrophy was defined as percentage difference of affected testis and the contralateral normal testis >50%.^14^ Paternity rate was only calculated among followed‐up patients older than 22 years since the legal marriage age in China is 22 years.

### 2.3 Surgical procedures and follow‐up

An urgent surgical scrotal exploration was performed for highly suspected TT. First, an upper scrotal incision was made after general anesthesia and then the skin and tunica vaginalis were incised to directly detect the torsion of testis. Second, the torsed testis was detorsed without tension; BCD was evaluated and 1% lignocaine was infiltrated within the spermatic cord. A small incision was made in the tunica albuginea to examine the blood supply and specify the Arda grade; simultaneously, a piece of moistened gauze with warm saline was used to cover the testicular tissue for 10–30 min. Third, the detorsed testis was re‐examined for potential salvageability. Then, a thorough consultation with the legal guardians was made during surgery to decide whether to perform the orchiectomy or just orchiopexy and whether to perform the CTF. The fixation of testes was conducted between the tunica albuginea and the tunica vaginalis with a non‐absorbable silk suture at two points. Finally, the surgery had been completed and the patient was under a strict observation. The operation records noted the direction of rotation, degree of cord twisting, BCD, communication results, surgical strategies and procedures.

A 1‐month, 3‐months, 6‐months, and 1‐year routine follow‐ups at the out‐patient clinic were suggested for those boys with TT after checking out and a immediate follow‐up was required when sudden scrotal symptoms were observed. After conceiving and designing this study, another extra follow‐up was arranged in June 2021. Patients were contacted by telephone, asked about scrotal symptoms, marriage and paternity status, and suggested a visit for clinical physical examination and ultrasound monitoring of the testes. Only the last follow‐up results were calculated if these patients had examined more than once.

### 2.4 Statistical methods

All the statistical analyses in this study were performed by the IBM SPSS Statistics for windows, version 25.0 (IBM Corp.). The Shapiro‐Wilk test was used to check the normal distribution of continuous parameters. Continuous variables were presented by median (interquartile range [IQR]) and categorical variables were presented as ratios (%). The *χ* ^2^ test and the Fisher's Exact test were used to compare dichotomous outcomes, and the Mann–Whitney *U* test was used to compare continuous outcomes without normal distribution. A *p*‐value <0.05 was considered statistically significant.

## 3 RESULTS

Initial medical database search yielded 315 patients with TT treated in our department, and only 140 cases conformed with our inclusion criteria. The median age of patients who underwent orchiopexy with and without CTF was 11.8 and 11.9 years, respectively (Table 1). The median age of patients who underwent orchiectomy with and without CTF was 11.1 and 13.0 years, respectively (Table 1). Both of these two groups were compared at age of surgery and laterality, and there was left predominance both in orchiopexy (81/102, 79.4%) and orchiectomy groups (30/38, 78.9%). However, those TT patients who underwent orchiopexy with CTF seemed to have a longer duration from symptoms onset to surgery (36 vs. 20 h, *p* = 0.046).

**TABLE 1:** Comparison of baseline characteristics in patients with testicular torsion between those who received CTF and those who did not.

	Accepted orchiopexy with CTF (*n*= 55)	Accepted orchiopexy without CTF (*n*= 47)	*p* value
Baseline characteristics			
Age at surgery (year), median (IQR)	11.8 (4.4, 13.7)	11.9 (6.8, 13.3)	0.752
Duration from symptoms onset to surgery (hour), median (IQR)	36.0 (12.0, 72.0)	20.0 (5.0, 48.0)	0.046*
Laterality, *n* (%)			0.410
Left	42 (76.4)	39 (83.0)	
Right	13 (23.6)	8 (17.0)	

	Accepted orchiectomy with CTF（*n*= 26）	Accepted orchiectomy without CTF (*n*= 12)	*p* value
Baseline characteristics			
Age at surgery (year), median (IQR)	11.1 (6.4, 13.4)	13.0 (5.4, 14.8)	0.322
Duration from symptoms onset to surgery (hour), median (IQR)	72.0 (44.0, 154.5)	90.0 (49.0, 170.0)	0.950
Laterality, *n* (%)			1.0
Left	20 (76.9)	10 (83.3)	
Right	6 (23.1)	2 (16.7)	

Abbreviations: CTF, contralateral testicular fixation; IQR, interquartile range.

*p ≤ 0.05.

As for intraoperative findings, patients who underwent orchiopexy with CTF seemed to have a higher rate of clockwise torsed testes compared with those who underwent orchiopexy only (*p* = 0.013). BCD was found in 15.7% of all torsed testes who underwent orchiopexy, 16.4% during contralateral testicular exploration, and only 10.9% in bilateral sides (Table 2). All resected testes were marked as Arda grade III. Added in total, only 2 BCDs in the affected side and 3 in the contralateral healthy side were found during surgery in patients who had orchiectomy (Table 3).

**TABLE 2:** Comparison of intraoperative findings and follow‐up results between patients with testicular torsion underwent salvage orchiopexy with or without CTF.

	Orchiopexy with CTF (*n* = 55)	Orchiopexy without CTF (*n* = 47)	*p* value
Intraoperative findings			
Direction of rotation, *n* (%)			0.013*
Clockwise	39 (70.9)	22 (46.8)	
Anticlockwise	16 (29.1)	25 (53.2)	
Grade of Arda, *n* (%)			0.272
I–II	38 (69.1)	37 (78.7)	
III	17 (30.9)	10 (21.3)	
Degree of cord twisting, *n* (%)			0.125
≤360°	28 (50.9)	31 (66.0)	
>360°	27 (49.1)	16 (34.0)	
Bell clapper deformity among exploration, *n* (%)			
Ipsilateral (affected) side	11 (20.0)	5 (10.6)	0.195
Contralateral (normal) side	9 (16.4)	‐	
Bilateral sides	6 (10.9)	‐	
Follow‐up results			
Age at follow‐up (year), median (IQR)	12.7 (5.1, 14.1)	12.4 (8.8, 14.0)	0.957
Follow‐up time (month), median (IQR)	5.0 (3.0, 19.0)	5.0 (2.0, 26.0)	0.788
Ipsilateral testicular atrophy, *n* (%)	38 (69.1)	22 (46.8)	0.023*
Contralateral testicular atrophy, *n* (%)	0 (%)	0 (%)	
Testicular volume (mL), median (IQR)			
Contralateral (normal) side	6.33 (1.15, 12.75)^a^	9.07 (2.18, 12.20)^b^	0.427
Age‐ and laterality‐matched healthy controls	3.66 (0.85, 7.99)^a^	6.04 (1.02. 9.99)^b^	‐
Total adverse events, *n* (%)	8 (14.5)	8 (17.0)	0.732
Subsequent orchiectomy, *n* (%)	2 (3.6)	4 (8.5)	0.410
Testicular tumor, *n* (%)	0 (0.0)	1 (2.1)	‐
Contralateral testicular torsion, *n* (%)	0 (0.0)	0 (0.0)	‐
Scrotal pain or discomfort, *n* (%)	6 (10.9)	3 (6.4)	0.501
Paternity rate among patients older than 22 years to data, *n*/*N* (%)	1/5 (20.0)	1/3 (33.3)	‐

Abbreviations: CTF, contralateral testicular fixation; IQR, interquartile range.

^a^ Comparison of testicular volume between contralateral testes of patients underwent orchiopexy with CTF and age‐ and laterality‐matched controls, *p* = 0.026.

^b^ Comparison of testicular volume between contralateral testes of patients underwent orchiopexy without CTF and age‐ and laterality‐matched controls, *p* = 0.041.

*p ≤ 0.05.

**TABLE 3:** Comparison of intraoperative and follow‐up findings between patients with testicular torsion who underwent orchiectomy with or without CTF.

	Orchiectomy with CTF (*n* = 26)	Orchiectomy without CTF (*n* = 12)	*p* value
Intraoperative findings			
Direction of rotation, *n* (%)			0.725
Clockwise	16 (61.5)	6 (50)	
Anticlockwise	10 (38.5)	6 (50)	
Grade of Arda, *n* (%)			‐
I–II	0 (0)	0 (0)	
III	26 (100)	12 (100)	
Degree of cord twisting, *n* (%)			1.0
≤360°	10 (38.4)	4 (33.3)	
>360°	16 (61.6)	8 (66.7)	
Bell clapper deformity among exploration, *n* (%)			
Ipsilateral (affected) side	2 (7.7)	0 (0)	‐
Contralateral (normal) side	3 (11.5)	0 (0)	‐
Bilateral sides	2 (7.7)	0 (0)	‐
Follow‐up results			
Age at follow‐up (year), median (IQR)	12.1 (7.9, 14.8)	15.0 (10.0, 19.3)	0.173
Follow‐up time (month), median (IQR)	5.0 (2.8, 36.0)	32.0 (8.5, 106.8)	0.017*
Testicular volume (mL), median (IQR)			
Contralateral (normal) side	8.94 (2.06, 15.51)^a^	2.34 (1.82, 13.46)^b^	0.508
Age‐ and laterality‐matched healthy controls	4.83 (0.98, 9.92)^a^	4.58 (0.84, 9.77)^b^	‐
Total adverse events, *n* (%)	1 (3.8)	5 (41.7)	0.008*
Testicular tumor, *n* (%)	0 (0)	0 (0)	‐
Contralateral testicular torsion, *n* (%)	0 (0)	1 (8.3)	‐
Scrotal pain or discomfort, *n* (%)	1 (3.8)	4 (33.3)	0.027*
Paternity rate among patients older than 22 years to data, *n*/*N* (%)	1/3 (33.3)	2/5 (40)	‐

Abbreviations: CTF, contralateral testicular fixation; IQR, interquartile range.

^a^ Comparison of testicular volume between contralateral testes of patients who underwent orchiectomy with CTF and age‐ and laterality‐matched controls, *p* = 0.040.

^b^ Comparison of testicular volume between contralateral testes of patients who underwent orchiectomy without CTF and age‐ and laterality‐matched controls, *p* = 0.381.

*p ≤ 0.05.

The median age of patients who underwent orchiopexy with and without CTF was 12.7 and 12.4 years, respectively, and the median follow‐up time was 5 months. Patients who had orchiopexy and CTF had a higher ipsilateral testicular atrophy rate compared with orchiopexy alone (69.1% vs. 46.8%, *p* = 0.023), while no contralateral testicular atrophy was detected in both groups. One patient who had left orchiopexy without CTF suffered ipsilateral testicular masses in 77 months after surgery, which was pathologically confirmed as mature teratoma by re‐exploration. One patient who had left orchiopexy was found with abdominal lymphoma 3 months after surgery. The median TV of the contralateral side of patients who underwent orchiopexy with CTF was 6.33 mL (IQR, 1.15, 12.75), a little smaller than patients who underwent orchiopexy alone of 9.07 mL (IQR, 2.18, 12.20), but did not reach the statistically significant difference (*p* = 0.427) (Table 2). Total adverse events, subsequent orchiopexy, and complained scrotal pain or discomfort were comparable between the two groups. There were one in five patients who had orchiopexy and CTF, and one in three orchiopexies who were more than 22 years of age already had children.

The median follow‐up time of patients who underwent orchiectomy without CTF was statistically longer than these patients with CTF (32 vs. 5 months, *p* = 0.017, Table 3). The median contralateral TV of patients who had orchiectomy with CTF and orchiectomy without CTF were 8.94 and 2.34 mL, respectively, but did not reach the significant difference (*p* = 0.508). One patient who underwent left orchiectomy without CTF experienced subsequent contralateral TT; fortunately, he had the urgent orchiopexy within 4 h and the remaining testicle had been rescued. Patients who had orchiectomy alone may have a higher risk of scrotal pain or discomfort compared with those who had orchiectomy with CTF (*p* = 0.027).

## 4 DISCUSSION

Due to the absence of evidence‐based guidelines, well‐designed comparative clinical studies, and long‐term follow‐up results regarding surgical strategies for TT, whether to perform the CTF when exploring the acute scrotum indeed creates a dilemma for the practicing pediatric urologists: on one hand, due to the fear of catastrophic bilateral testicular loss, and on the other hand, due to the fear of direct iatrogenic injury or indirect potential damage to the contralateral healthy testicle.^8^, ^9^ According to the currently available literature, the decision for CTF in clinical scenarios is mainly based on surgeon's experiences and parents' counseling.^8^

In order to fill up a stone for this lacking knowledge gap, we conducted this study to explore the possible advantages and disadvantages of CTF in pediatric TT. According to our study, patients who underwent orchiopexy with CTF had comparable contralateral TV, subsequent orchiectomy rate, scrotal pain or discomfort rate, and total adverse events, compared with those patients who underwent orchiopexy only, and no any contralateral testicular atrophy was detected. We presumed that CTF did not bring obvious harm to the contralateral testes and also may not support the contralateral testes. Our findings showed that patients with orchiopexy and CTF had a higher ipsilateral testicular atrophy rate than orchiopexy alone; this should be attributed to the longer duration time from symptoms onset to surgery in patients with orchiopexy and CTF, as we know, time duration and degree of cord twisting are the two main determinants for testicular salvage after torsion.^15^, ^16^ The longer the duration was, the heavier the degree of testicular damage was. As a result, parents were more likely to choose the CTF for fearing of contralateral testicular injury after a thorough explanation of the advantages and disadvantages of CTF, and in particular, the increased risk of ipsilateral atrophy, for those patients with longer duration from symptoms onset to surgery.

Patients who underwent orchiectomy only seemed to have a higher risk of subsequent scrotal symptoms than those patients who had simultaneous orchiectomy and CTF. We still could not interpret these data well. Patients who underwent orchiectomy only had a much longer follow‐up time, which may be because these patients have more frequent scrotal symptoms and feel less relieved concern about the contralateral testis, compared with those patients who already had fixed. Generally speaking, CTF had no serious bad effects on the contralateral testicle and no obvious potential protective contribution for the contralateral testes for these patients who underwent orchiectomy was observed; robust evidence was still greatly needed.

BCD is associated with an increased risk of TT. According to our study data extracted from reliable operation records, only 15.7% of all torsed testes underwent orchiopexy found with BCD, 16.4% of contralateral testes, and 10.9% in bilateral sides, which were much lower than as high as 78% reported by Martin and colleagues in 2014.^2^ In addition, Martin et al. found only one case of partial contralateral BCD in 50 perinatal TT children, while 21 out of 27 contralateral testes had BCD in older boys with acute TT, indicating that the incidence of BCD in TT patients still varied a lot in different populations. Though the etiology of TT may be closely associated with BCD, BCD contributed little for diagnosis and treatment of TT, because BCD was usually found during surgery.

All surgical interventions should be weighed against the possible advantages and potential injury, especially in pediatrics. In the decision of CTF, apart from surgeon's experiences, the legal guardians of TT boys should be thoroughly counseled on the advantages and disadvantages of each surgical procedure and long‐term implications after surgery to gain better understanding from the victims. Moore and colleagues conducted the latest systematic review on surgical techniques of TT, contralateral testes were fixed in 57.6% of all cases, and no episodes of ipsilateral retorsion were reported.^7^ Most interestingly, Alnadhari et al. reported a recurrent TT after previous fixation and emphasized the use of nonabsorbable suture in at least two points to fix the testicle.^17^ Subsequent or recurrent ipsilateral or contralateral TT was very rare among literatures, which was the most important immediate consciousness of possible TT and timely actions for salvage.

There is no doubt that still some limitations are noteworthy in our study. First, the retrospective design of this study may have inevitable recall bias and selection bias, the judgment of scrotal symptoms by patients or parents through a phone call may not be very accurate, and these patients who have discomfort more likely have a follow‐up. Second, the short‐term complications, such as scrotal swelling, wound infection, and scrotal abscess, were not evaluated in this study due to the short‐term complications that are greatly associated with surgical procedures and surgeon skills. Third, the follow‐up time was not long enough and the rate of lost to follow‐up was relatively high; the patients were not consecutively and regularly followed. Fourth, the sample size was relatively small, especially for patients who underwent orchiectomy, and the TV, adverse events, and paternity rate used in this study may not be ideally present as the comparison parameters between orchiopexy and orchiectomy with or without CTF. Finally, the terminal functional evaluation of testes as well as exocrine and endocrine functions were not assessed to compare the spermatogenic functions and endocrine productions after TT.

## 5 CONCLUSION

Contralateral TV, adverse events, and paternity rates were compared between patients who underwent orchiopexy with or without CTF during follow‐up. CTF would not induce TV loss, testicular atrophy, or scrotal pain. Contralateral testicular discomfort and even subsequent TT seem to be more easily observed during follow‐up in patients without CTF. As a consequence, we still recommend routine surgical fixation of contralateral testicle during surgery for patients with TT.

## AUTHOR CONTRIBUTIONS

Chengjun Yu and Shengde Wu conceived and designed this study. Chengjun Yu and Yi Wei extracted the data. Chengjun Yu, Qinlin Shi, and Sheng Wen led analysis and interpretation of data, drafted the manuscript, and revised content based on feedback. Tao Lin, Dawei He, and Guanghui Wei assisted with the conception and design, interpretation of data, and critical revision of drafts. Shengde Wu provided funding support and provided critical revision of drafts.

## CONFLICT OF INTEREST STATEMENT

Shengde Wu is an associate editor of Pediatric Discovery. To minimize bias, he was excluded from all editorial decision‐making related to the acceptance of this article for publication. The other authors declare no conflict of interests.

## ETHICS STATEMENT

The local institutional review board of Children's Hospital of Chongqing Medical University approved this study (File No. 2021‐384).

## CODE AVAILABILITY

Not applicable.

## CONSENT TO PARTICIPATE

A general written informed consent was obtained from their legal guardians during hospitalization.

## CONSENT FOR PUBLICATION

All the authors approved the final version of this manuscript and agreed for possible publication.

## Data Availability

All data are available from the corresponding author upon reasonable request.
